# Editorial: Bacterial transcription factors and the cell cycle, volume II

**DOI:** 10.3389/fmicb.2023.1252924

**Published:** 2023-07-25

**Authors:** Morigen, Monika Glinkowska, Jianping Xie, Richa Priyadarshini, Kazutoshi Kasho

**Affiliations:** ^1^State Key Laboratory of Reproductive Regulation and Breeding of Grassland Livestock, School of Life Sciences, Inner Mongolia University, Hohhot, China; ^2^Department of Bacterial Molecular Genetics, University of Gdansk, Gdańsk, Poland; ^3^Institute of Modern Biopharmaceuticals, School of Life Sciences, Southwest University, Chongqing, China; ^4^Department of Life Sciences, School of Natural Science, Shiv Nadar Institution of Eminence, Gautam Buddha Nagar, India; ^5^Department of Molecular Biology, Graduate School of Pharmaceutical Sciences, Kyushu University, Fukuoka, Japan

**Keywords:** bacteria, transcription factor, transcription, signaling, cell division

The progression of the bacterial cell cycle is a result of the fluctuation in gene expression regulated by transcription factors. It is well-known that the initiator protein DnaA controls gene expression as a transcription factor (Messer and Weigel, 1997) while it triggers the initiation of DNA replication (Fuller and Kornberg, 1983). Thus, it is reasonable to assume that dual functional DnaA temporally and spatially coordinates different cellular pathways with the initiation of DNA replication. Indeed, in *Caulobacter crescentus*, DnaA couples chromosome replication with the expression of GcrA and CtrA, which are two important oscillating regulators. The regulatory cascade made of DnaA/GcrA/CtrA pushes forward the succession of the cell cycle coordinately (Collier et al., 2006). As reviewed recently (Menikpurage et al., 2021), DnaA might be involved in the development of different cellular events of quorum sensing, cell motility, DNA repair (Wurihan et al., 2018), and cell cycle control by regulating the expression of the genes associated. Most recently, DnaA was reported to regulate transcription attenuation of the *his* operon (Yao et al., 2023). Other transcription factors, for example, RpoS in *Coxiella* controls the expression of the developmental cycle genes (Moormeier et al., 2019), and BolA in the Gram-negative bacteria turns on biofilm development while it turns off motility as a transcription factor (Dressaire et al., 2015). In Volume I of the Research Topic (Morigen et al., 2021), the following studies were included: (i) functional identification of transcription factors Mfd, DagR, RsbW homologs, Crp1, OxyR, and NprR in various bacteria; (ii) determination of histone-like nucleoid-binding protein YbaBCc and genome-wide cell cycle-dependent binding patterns of IHF to chromosome with base-pair resolution using GeF-seq; (iii) direct interaction and function of ZapE with FtsZ in cell division and the DnaA (L366K)-mediated restoration of growth defect due to the accumulation of lipoprotein Lpp (C21G). Also, the orisome assembling and function in each cell cycle and the conserved location of CtrA phosphorelay associated genes to *ori* and *ter* of the bacterial circular chromosome were described. Volume II of the Research Topic is also a collection of articles focusing on the bacterial transcription factors and the cell cycle, particularly transcription factors, transcription, and cell division.

## Transcription factors and transcription

The OmpR protein is a transcription factor and also a part of the EnvZ/OmpR two-component system (TCS), which has been shown to be involved in prodigiosin biosynthesis. Prodigiosin is a secondary metabolite that has various pharmacological activities. Prodigiosin production has been found in some bacteria and is largely produced in *Serratia marcescens*. The biosynthesis of prodigiosin in *S. marcescens* is affected by temperature, pH, and medium composition and regulated by the EnvZ/OmpR system, which senses various environmental stress and growth conditions. Jia et al. showed that OmpR increased prodigiosin production in *S. marcescens* FZSF02. Further, in this study, OmpR was found to directly bind to the promoter regions of the *pig* gene cluster that is associated with prodigiosin biosynthesis and of the *envZ*/*ompR* genes. The authors also found that the 5′CATTTATTTACATTTAC3′ sequence in the *pig* promoter was the binding target of OmpR by DNase I footprinting assay. The work concludes that OmpR regulates its own expression and the *pig* gene cluster, and subsequently governs the production of prodigiosin in *S. marcescens*, being a transcription factor. Another transcription factor, Ste12, has been shown to regulate stress tolerance and sexual reproduction in fungi. Lyu et al. constructed the phylogenetic tree of Ste12-like proteins of *Flammulina filiformis* and other fungi by comparison of the amino acid sequences and showed that the Ste12-like proteins contained the conserved amino acid sequences with three typical motifs, namely, motif 1, 2, and 3. Subsequently, using *Agrobacterium tumefaciens*-mediated transformation, Lyu et al. constructed four *ste12*-like overexpression transformants of *F. filiformis*, and it was found that these overexpression transformants were more tolerant to salt, cold, and oxidative stress with an increased number of fruiting bodies but at a slow growth rate. The results allow the authors to conclude that the Ste12-like protein participates in the regulation of abiotic stress tolerance and fruiting body development in *F. filiformis*.

Interestingly, in the WalK/WalR two-component system of *Streptococcus mutans*, Kong et al. found that the histidine kinase WalK had an extended C-terminal tail (CTT) in 14 different TCSs, and CTT plays a crucial role in the interaction of WalK with its response regulator WalR, a transcription factor. It was demonstrated that the tryptophan in CTT was required for WalK signaling since W443 in the CTT is essential for the phosphotransferase and phosphatase activities of WalK. Any mutation which disrupts the interaction of WalK with WalR in CTT might impair the signaling processes of this TCS. The tryptophan was also shown to be key for WalK to compete with a DNA that contains a WalR binding motif and is important for transcription control *in vivo* and biofilm formation. Similarly, the *Staphylococcus aureus* WalK has a remarkable CTT although it is short in length; it possesses a conserved W-acidic motif. In conclusion, the W-acidic motif in WalK is essential for the WalK-WalR interaction, playing a key role in the WalR/WalK-dependent signal transduction, biofilm formation, and transcription regulation. It should be noted that a transcriptome analysis on an isolate of *Cellulomonas fimi* from polluted river water, strain Clb-11, is interesting. The Clb-11 strain could generate electricity in microbial fuel cells with carboxymethyl cellulose as the carbon source and secrete chromate reductase or electron mediator to reduce Cr (VI) to Cr (III). To understand how the Clb-11 strain reduces Cr (VI) to Cr (III), Cao et al. analyzed differentially-expressed genes involved in different Cr (VI) stress responses in Clb-11 by RNA-sequencing and found that 99 genes were up-regulated while 78 genes were down-regulated as the Cr (VI) concentration increased. The differentially-expressed genes are mainly associated with DNA repair, ABC transporters, amino sugar, and carbon metabolism to cope with Cr (VI) stress-induced DNA damage and cellular toxic effect. The work may provide clues to understanding the molecular mechanism of Cr (VI) reduction in the Clb-11 strain.

## Cell division

Bacterial cell division is a highly regulated molecular process in which the conserved tubulin homolog FtsZ assembles into the Z-ring at mid-cell on the interior of the cell membrane through polymerization. The FtsZ polymers recruit FtsA in *Escherichia coli*, a membrane-associated protein, by directly interacting with it. FtsA polymerizes into an actin-like structure, promoted by ATP binding, displaying considerable flexibility in conformation across different nucleotide-bound states. Morrison et al. showed that several amino acid residues near the nucleotide-binding site in FtsA were key for function, being associated with ATP hydrolysis, phospholipid (PL) binding, ATP-dependent vesicle remodeling, and recruitment to the divisome *in vivo*. For example, Ser-84 and Glu-14 residues in FtsA are essential for ATP-dependent vesicle remodeling and magnesium-dependent membrane release of FtsA from vesicles *in vitro*; FtsA (A188V) is defective for rapid ATP hydrolysis and ATP-dependent remodeling of PL vesicles *in vitro*. The work concludes that nucleotide-dependent activities of FtsA regulate the early establishment of a functional divisome during the *E. coli* cell cycle.

## Author contributions

M and JX wrote the first manuscript draft. M edited the final version. All authors listed have made a substantial, direct, and intellectual contribution to the work, revised, and approved it for publication.

## References

[B1] Collier J. Murray S. R. Shapiro L. (2006). DnaA couples DNA replication and the expression of two cell cycle master regulators. Embo J. 25, 346–356. 10.1038/sj.emboj.7600927 16395331 PMC1383511

[B2] Dressaire C. Moreira R. N. Barahona S. Alves De Matos A. P. Arraiano C. M. (2015). BolA is a transcriptional switch that turns off motility and turns on biofilm development. mBio 6, e02352–02314. 10.1128/mBio.02352-14 25691594 PMC4337573

[B3] Fuller R. S. Kornberg A. (1983). Purified dnaA protein in initiation of replication at the *Escherichia coli* chromosomal origin of replication. Proc. Natl. Acad. Sci. U S A. 80, 5817–5821. 10.1073/pnas.80.19.5817 6310593 PMC390166

[B4] Menikpurage I. P. Woo K. Mera P. E. (2021). Transcriptional activity of the bacterial replication initiator DnaA. Front. Microbiol. 12, 662317. 10.3389/fmicb.2021.662317 34140937 PMC8203912

[B5] Messer W. Weigel C. (1997). DnaA initiator–also a transcription factor. Mol. Microbiol. 24, 1–6. 10.1046/j.1365-2958.1997.3171678.x 9140960

[B6] Moormeier D. E. Sandoz K. M. Beare P. A. Sturdevant D. E. Nair V. Cockrell D. C.. (2019). Coxiella burnetii RpoS regulates genes involved in morphological differentiation and intracellular growth. J. Bacteriol. 4, 201. 10.1128/JB.00009-19 30745369 PMC6436347

[B7] Morigen Glinkowska, M. Xie J. (2021). Editorial: bacterial transcription factors and the cell cycle. Front Microbiol. 12, 821394. 10.3389/fmicb.2021.821394 35003050 PMC8739904

[B8] Wurihan Gezi Brambilla E. Wang S. Sun H. Fan L. Shi Y. Sclavi B. Morigen (2018). DnaA and LexA proteins regulate transcription of the *uvrB* Gene in *Escherichia coli:* the role of DnaA in the control of the SOS regulon. *Front. Microbiol* .9, 1212. 10.3389/fmicb.2018.01212 29967594 PMC6015884

[B9] Yao Y. Sun H. Wurihan G. Gezi S. K Fan L. Morigen. (2023). A DnaA-dependent riboswitch for transcription attenuation of the his operon. mLife 2, 126–140. 10.1002/mlf2.12075 PMC10989985 38817620

